# Supplementary material for: Experimental evolution of a pathogen confronted with innate immune memory increases variation in virulence
Source: PLoS Pathog. 2025 Jun 18;21(6):e1012839. doi: 10.1371/journal.ppat.1012839 (PMC12176410; doi:10.1371/journal.ppat.1012839)
Supplement: S1 Table — (DOCX) [file ppat.1012839.s001.docx]

**Table 1** qPCR primers and their amplification efficiencies

| gene | forward primer | reverse primer | efficiency |
| --- | --- | --- | --- |
| *Cry3a* | AACAGATGAAGCAAGTACACAAACG | CTGTTGTTTCTGGAGGCAATTGATC | 105% |
| *Yqey* | AGCTGGTCGTGAAGACCTTG | CGGCATAACAGCAGTCATCA | 94% |
| *rps21* | AAATCTGAAGCGGCAAGAA | AAGATCGGTTTCTAAACTGGTACA | 102% |
