# Supplementary material for: Experimental evolution of a pathogen confronted with innate immune memory increases variation in virulence
Source: PLoS Pathog. 2025 Jun 18;21(6):e1012839. doi: 10.1371/journal.ppat.1012839 (PMC12176410; doi:10.1371/journal.ppat.1012839)
Supplement: S2 Table — Btt- and Bt407- do not carry the Cry3a gene. Priming and control lines were evolved from ancestral strain. Mean ΔCt values by subtracting the target gene Ct value from the geometric mean of the Ct values of the housekeeping genes. (DOCX) [file ppat.1012839.s002.docx]

**Table 2** Relative expression of *Cry3a* gene in cultures from different *Btt* strains. *Btt-* and *Bt407-* do not carry the *Cry3a* gene. Priming and control lines were evolved from ancestral strain. Mean ΔCt values by subtracting the target gene Ct value from the geometric mean of the Ct values of the housekeeping genes.

| bacterial culture | mean ΔCt | std |  |  |  |
| --- | --- | --- | --- | --- | --- |
| Btt- | -11.19 | 3.69 |  |  |  |
| Bt407- | -10.15 | 0.57 |  |  |  |
| priming | 0.62 | 1.38 |  |  |  |
| control | 1.01 | 1.31 |  |  |  |
| ancestral | 1.77 | 1.31 |  |  |  |
