# Supplementary material for: Experimental evolution of a pathogen confronted with innate immune memory increases variation in virulence
Source: PLoS Pathog. 2025 Jun 18;21(6):e1012839. doi: 10.1371/journal.ppat.1012839 (PMC12176410; doi:10.1371/journal.ppat.1012839)
Supplement: S3 Table — Shown are the results of linear mixed effects model with block as experimental factor. Significant differences are shown in bold type face. (DOCX) [file ppat.1012839.s003.docx]

**Table 3** Differences of *Cry3a* gene expression of individual evolved lines compared to the ancestral strain. Shown are the results of linear mixed effects model with block as experimental factor. Significant differences are shown in bold type face.

| line | estimate | s. e. | dF | t-value | p-value |
| --- | --- | --- | --- | --- | --- |
| intercept | 1.77 | 0.52873 | 5.62228 | 3.342 | 0.017 |
| C1 | -0.56 | 0.59026 | 50 | -0.947 | 0.348 |
| **C2** | **-1.23** | 0.59026 | 50 | -2.087 | **0.042** |
| C3 | 0.25 | 0.59026 | 50 | 0.421 | 0.675 |
| C4 | 0.52 | 0.59026 | 50 | 0.878 | 0.384 |
| **C5** | **-2.34** | 0.59026 | 50 | -3.958 | **<0.001** |
| **C6** | **-1.52** | 0.59026 | 50 | -2.573 | **0.013** |
| C7 | 0.31 | 0.59026 | 50 | 0.525 | 0.602 |
| **C8** | **-1.48** | 0.59026 | 50 | -2.514 | **0.015** |
| P1 | -0.04 | 0.59026 | 50 | -0.062 | 0.951 |
| P2 | -0.71 | 0.59026 | 50 | -1.203 | 0.235 |
| P3 | -0.83 | 0.59026 | 50 | -1.412 | 0.164 |
| P4 | -0.74 | 0.59026 | 50 | -1.26 | 0.213 |
| **P5** | **-2.44** | 0.59026 | 50 | -4.136 | **<0.001** |
| **P6** | **-3.52** | 0.59026 | 50 | -5.964 | **<0.001** |
| **P7** | **-1.52** | 0.59026 | 50 | -2.575 | **0.013** |
| P8 | 0.65 | 0.59026 | 50 | 1.101 | 0.276 |
