# Supplementary material for: Experimental evolution of a pathogen confronted with innate immune memory increases variation in virulence
Source: PLoS Pathog. 2025 Jun 18;21(6):e1012839. doi: 10.1371/journal.ppat.1012839 (PMC12176410; doi:10.1371/journal.ppat.1012839)
Supplement: S1 Fig — (DOCX) [file ppat.1012839.s004.docx]

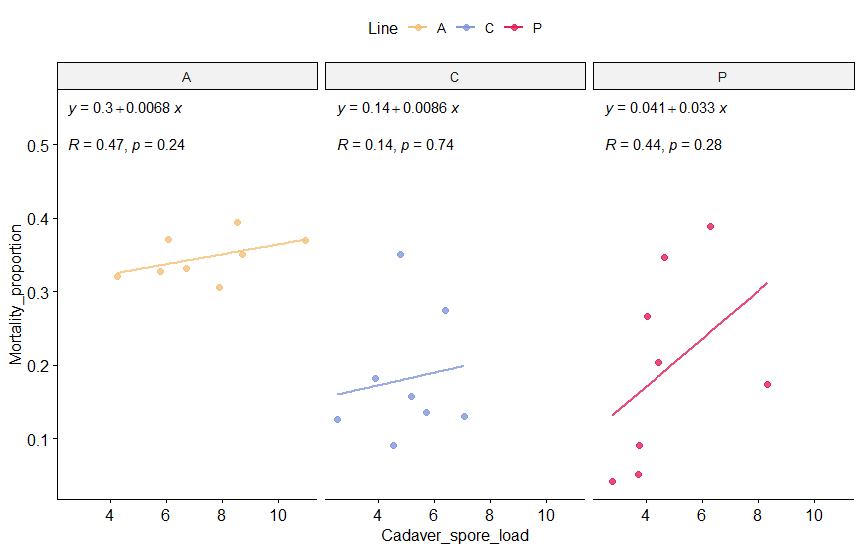


**Figure** **S1.** Correlation between spore load and virulence in ancestral line (A), control line(C) and primed line (P) in primed hosts
