# Supplementary material for: Experimental evolution of a pathogen confronted with innate immune memory increases variation in virulence
Source: PLoS Pathog. 2025 Jun 18;21(6):e1012839. doi: 10.1371/journal.ppat.1012839 (PMC12176410; doi:10.1371/journal.ppat.1012839)
Supplement: S3 Fig — ΔCt values were calculated by subtracting the Ct value of the target gene, Cry3A from the geometric mean of the Ct values of two housekeeping genes (Yqey and rps21). (DOCX) [file ppat.1012839.s006.docx]

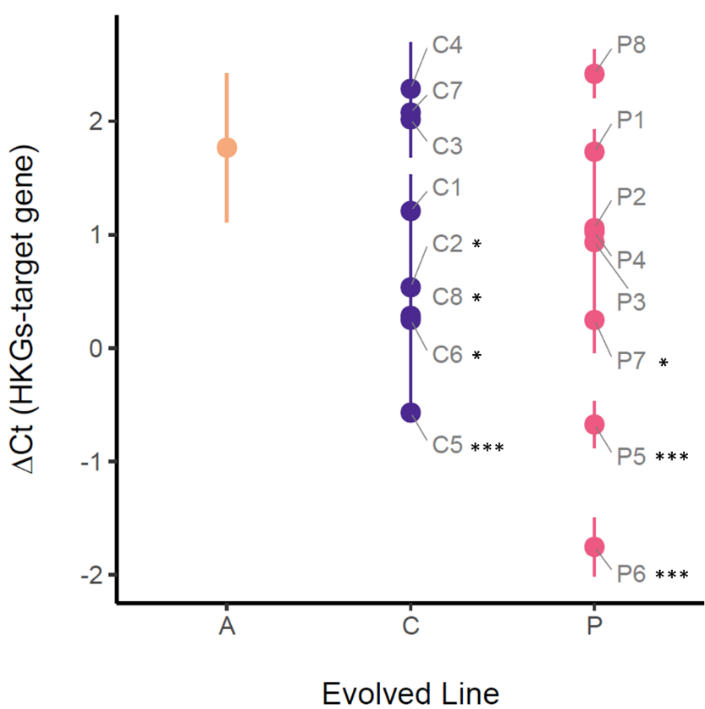


**Figure S3.** Relative expression of *Cry3a* gene for evolved lines. ΔCt values were calculated by subtracting the Ct value of the target gene, *Cry3A* from the geometric mean of the Ct values of two housekeeping genes (*Yqey* and *rps21* ). Per evolved line and ancestral strain, the mean and standard error for four replicates are given. Asterisks indicate significant differences to the ancestral strain (*= p-value <0.05, ***= p-value <0.0001
