# Supplementary material for: Experimental evolution of a pathogen confronted with innate immune memory increases variation in virulence
Source: PLoS Pathog. 2025 Jun 18;21(6):e1012839. doi: 10.1371/journal.ppat.1012839 (PMC12176410; doi:10.1371/journal.ppat.1012839)
Supplement: S4 Fig — A = ancestral, C = control evolved, P = primed evolved. (DOCX) [file ppat.1012839.s007.docx]

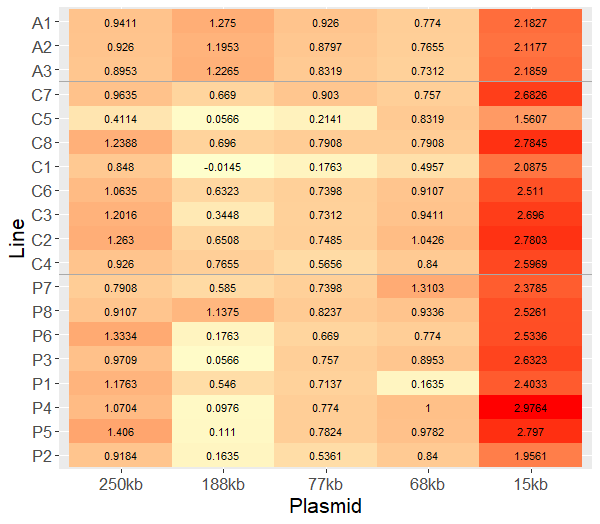


**Figure S4:** Heatmap for the Log2 transformed values of plasmid coverage divided by chromosome coverage for each replicate line. A = ancestral, C = control evolved, P = primed evolved. The evolved lines are ordered by evolution treatment and by virulence.
