# Supplementary material for: Experimental evolution of a pathogen confronted with innate immune memory increases variation in virulence
Source: PLoS Pathog. 2025 Jun 18;21(6):e1012839. doi: 10.1371/journal.ppat.1012839 (PMC12176410; doi:10.1371/journal.ppat.1012839)
Supplement: S5 Fig — (DOCX) [file ppat.1012839.s008.docx]

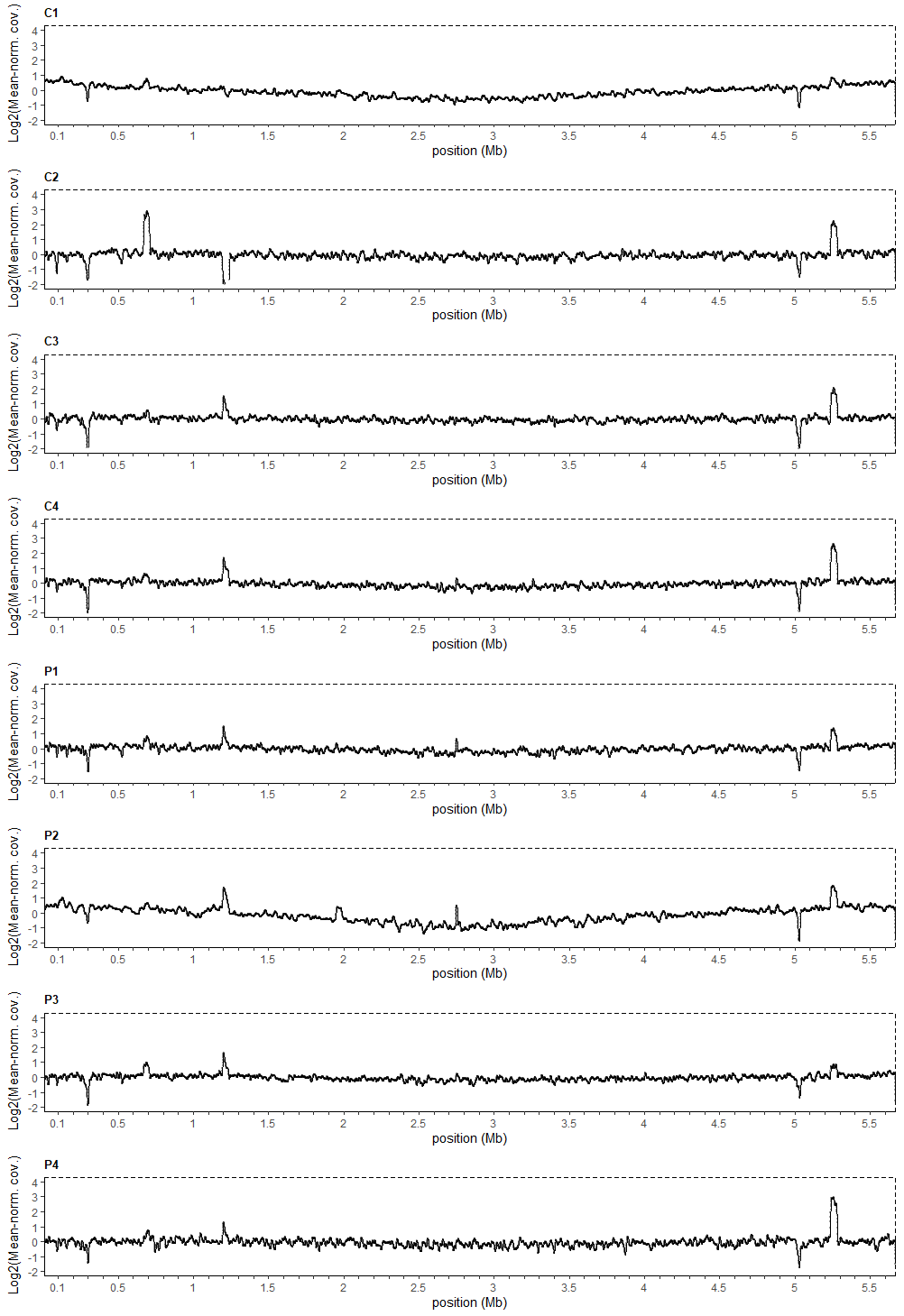


**Figure S5:** Exemplary log2 transformed mean-normalized coverage plots of reads across the chromosome for control (C1 – 4) and priming (P1 – 4) evolved lines.
