# Supplementary material for: Experimental evolution of a pathogen confronted with innate immune memory increases variation in virulence
Source: PLoS Pathog. 2025 Jun 18;21(6):e1012839. doi: 10.1371/journal.ppat.1012839 (PMC12176410; doi:10.1371/journal.ppat.1012839)
Supplement: S6 Fig — (DOCX) [file ppat.1012839.s009.docx]

**
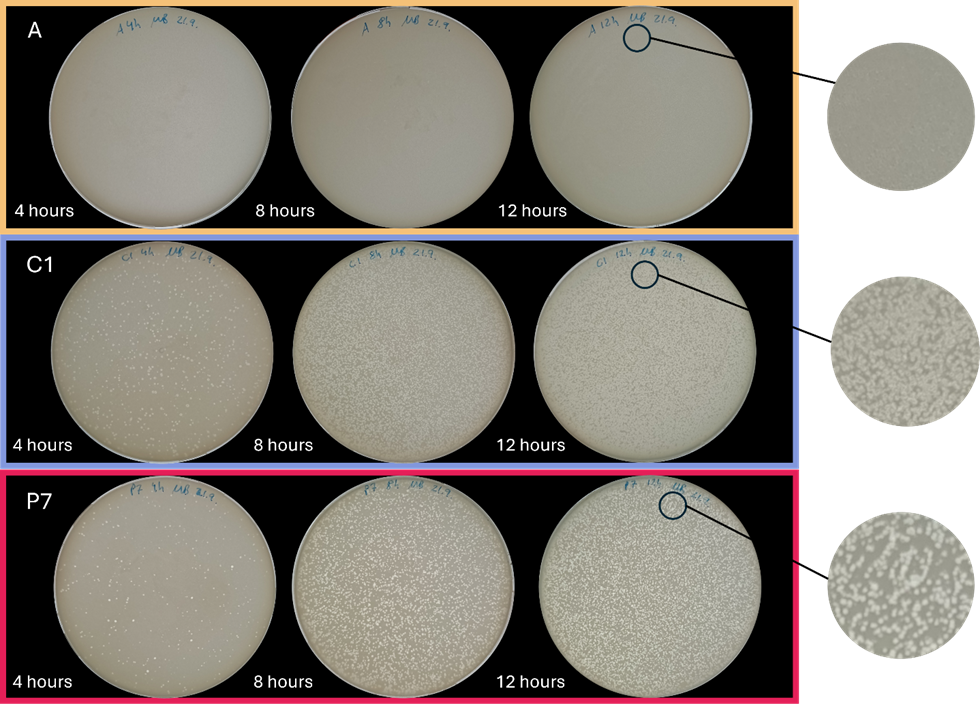
**

**Figure S6:** Double layer agar assays of an ancestral line A and evolved lines C1 (control evolved) and P7 (priming evolved). Culture samples were taken after 4 hours, 8 hours and 12 hours of growth in LB medium.
