# Supplementary material for: Experimental evolution of a pathogen confronted with innate immune memory increases variation in virulence
Source: PLoS Pathog. 2025 Jun 18;21(6):e1012839. doi: 10.1371/journal.ppat.1012839 (PMC12176410; doi:10.1371/journal.ppat.1012839)
Supplement: S7 Fig — Correlated traits include all phenotypic traits with active mobile elements (A, B) or Fitness and Priming sensitivity values with active mobile elements (C, D). (DOCX) [file ppat.1012839.s010.docx]

**
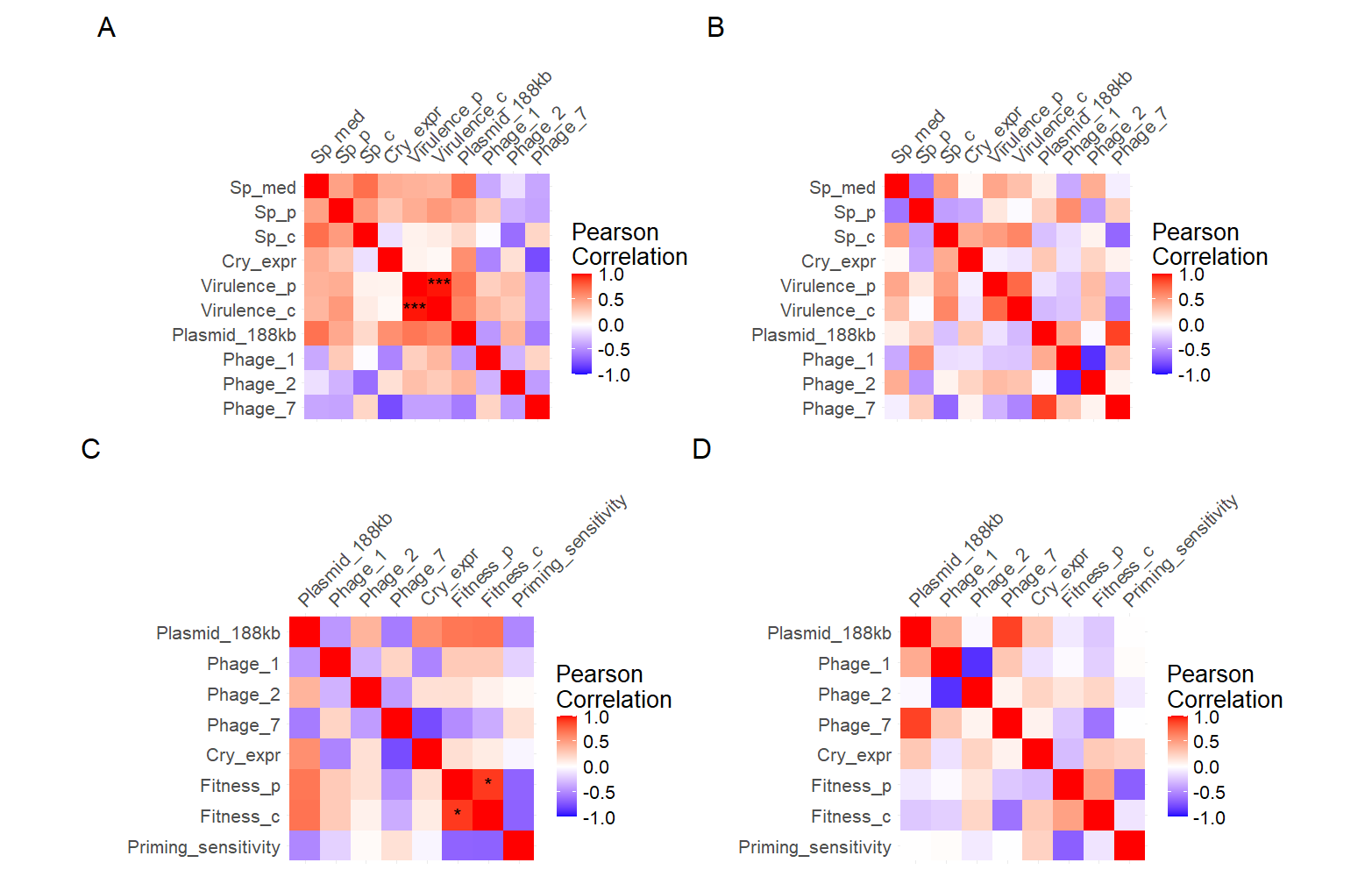
**

**Figure S7:** Pearson correlation matrix for the primed evolved pathogen (A, C) and the control evolved pathogen (B, D). Correlated traits include all phenotypic traits with active mobile elements (A, B) or Fitness and Priming sensitivity values with active mobile elements (C, D). Asterix indicates which correlations were statistically significant (FDR adjusted p value < 0.05). None of the correlations were statistically different between the primed and control evolved pathogen (BH adjusted p value < 0.05).
