# Supplementary material for: Experimental evolution of a pathogen confronted with innate immune memory increases variation in virulence
Source: PLoS Pathog. 2025 Jun 18;21(6):e1012839. doi: 10.1371/journal.ppat.1012839 (PMC12176410; doi:10.1371/journal.ppat.1012839)
Supplement: Attachment — Submitted filename: PPATHOGENS-D-24-02760_reviewer_AMDreport.docx [file ppat.1012839.s011.docx]

Response to Authors:

**Summary**:

In this study, Korša et al asked whether immune priming in the insect host Tribolium castaneum result in the evolution of increased virulence in the entomopathogenic bacteria Bacillus thuringiensis tenebrionis (Btt). Using a one-sided experimental evolution set-up, they subject Btt to undergo selection pressure in either an immune primed host or non-primed host for 8 selection cycles. To test the effects of their selection treatments, they performed a common garden experiment where evolved-lines of Btt and the ancestral strain were phenotyped for host-mortality (virulence) and spore load (transmission) in either an immune primed host or non-primed host environment. The authors observed that both evolved Btt lines did not increase virulence or transmission, in-fact they were reduced, when infecting primed or non-primed hosts. Interestingly, they found that the selection pressure of immune priming resulted in increased variation in Btt virulence. Genomic sequencing of these evolved strains revealed, when compared to the ancestral strain, minimal chromosomal mutations and reduced covered of the plasmid 188 kb, known to carry genes encoding virulence factors like the Cry toxin. The authors find some evidence that the evolved Btt lines have increased lytic phage activity, which could potentially explain reduced sporulation in those lines. While some additional experiments can help complete the presented work, this is an interesting study that interrogates whether incomplete immunity like immune priming can act as a selection pressure for bacteria pathogens to evolve virulence mechanisms against. The study appears mostly sound; however, the authors should revise the language to improve readability.

**Major Comments**

1. What prior literature are the authors using to base their hypothesis that immune priming would evolve for higher virulence in *Btt*? They cite Hoang, *et al* (2024) and Rafaluk *et al* (2017) as examples of evolved higher virulence, but they also cite evidence from Ford, *et al* (2016) that pathogens evolve lower virulence. Mikonranta et al (2015, BMC Ecology and Evolution, https://doi.org/10.1186/s12862-015-0447-5) show that one-side evolution of *Serratia marcescens* DB11 in *D. melanogaster* resulted in reduced virulence, which the authors did not cite. It would be helpful to clarify what aspects from the literature motivate the authors hypothesis that *Btt* evolves higher virulence in response to immune priming.
2. In the second paragraph of the discussion section, the authors discuss three possible explanations to justify the observed lower virulence of evolved *Btt* lines: 1) evolution of high virulence is costly in the absence of co-evolution, 2) virulence factors encoded on mobile elements like plasmids are costly, 3) virulence evolution could be reduced due to other microbes in the host environment. While the authors do a great job discussing point two, the description of points one and three are minimal and could be expanded on what aspects of their study correspond with these points they cite? Additionally, in point 2, the authors discuss Cry toxins do not play a critical role for *Btt* virulence in beetles, so why focus on measuring *Cry3Aa* expression over other virulence factors? Were there any mutations observed in virulence factors like chitinases or proteases, or does plasmid 188kb encode additional virulence genes? Using the existing sequencing results, I think the current study would benefit from the addition of reporting of how other virulence factors were impacted in the evolved lines.
3. The potential increase in lytic phage activity in both evolved lines in *Btt* is interesting and I think could be expanded on in this study. In the double layer agar experiments, the ancestral strain acts as a negative control since it does not have active phages, so repeating this experiment and the other phenotypes measured (mortality and sporulation) with a *Btt* strain known to have active phages can be a positive control. Thus, if a *Btt* line with active phages show similar phenotypes to the evolved strains, potentially, this increased lytic activity maybe a mechanism for reduced virulence. Also, is there a difference in the number of phages between immune primed and non-immune primed evolved strains? The double-layer agar assay is quantifiable, so it should be feasible to score the number of phages to test whether the priming evolved and non-primed evolved strains differ.
4. One of the major findings in this paper is that evolution to immune priming results in increased variation to virulence, however, the authors only show host mortality as the only phenotype to demonstrate this. Increased host mortality is associated with higher virulence phenotypes in pathogens. Since the authors found that *Btt* evolved in beetle larvae exhibit reduced virulence, I think the study could be complemented by including at least one or two other disease phenotypes associated with virulence. For example, measuring *Btt* pathogen load during the course of infection or measure pupation rate of larvae that survive acute infection.

**Minor Comments**

Here, I list comments specific to each section of the manuscript.

**Introduction**:

- More detail on what the authors define as varying levels of virulence would provide welcome context here. For example, line 42 discusses intermediate levels of virulence of the *Myxoma* virus without describing exactly what intermediate virulence looks like in contrast to high. This could also be revisited in the discussion since the authors did not find evidence for immune primed evolved lines to increase virulence, potentially *Btt* evolved intermediate virulence mechanisms.
- More information on the activated immune response of primed *Tenebrio castaneum* vs an active immune response in infected, non-primed larvae would be additional context. For example, description of what are the primary effectors (e.g. antimicrobial peptides, ROS, immune cells) of *T. castaneum* that are upregulated after infection and priming and which aspect of the response primarily control *Btt* infection. Additionally, more information on the *Btt* infection cycle in larvae in the wild would-be helpful context such as including how larvae would encounter spores in nature and how spores reproduce and disperse from larvae cadavers.
- The authors briefly discuss how external host immunity (line 63) and defensive microbes (line 64) are examples of factors that can impact pathogen virulence without much detail on how they define these terms. For clarity, it would be helpful to provide additional context on the type of pathogen and the infecting host from these cited studies is helpful to know because unique properties of different pathogen species and their distinct host environment could influence what virulence strategies they evolve.
- Do the authors consider transmission as a form of virulence? I believe they are connected, but the authors should clarify what makes virulence and transmission distinct.

**Results**:

“Does host immune priming affect pathogen virulence evolution?” Section

Figure 1:

- What is the motivation or prior work used to justify performing 8 selection cycles, and how many generations of *Btt* (doubling time) occur during infection? The papers they cite like Hoang, et al (2024) performed 14 selection passages on *P. aeruginosa* and Ford, et al (2016) passaged *Enterococcus faecalis* and *Staphylococcus aureus* for 10 passages.
- For clarity, I think it is helpful to have a prescribed naming convention for the immune primed evolved *Btt* and the non-primed (author refers to control) evolved *Btt* to be distinct from the immune primed host environment and the control host environment.

Figure 2:

- Line 142-143: Here the authors are trying to directly compare average mortality between control hosts and primed hosts infected with ancestral, immune primed, and non-immune primed *Btt*. I think the data across these two figures should be compiled in the same graph if they are going to make direct make the comparisons between the two host environments.
- Line 145: What makes this analysis different than the one performed above? I'd explicitly state what type of model you created in the body of the results and what are the major factors being tested.
- Line 156: The authors state “the primed host, we observed higher variance in virulence across primed-evolved replicates (σ2 = 0.862) than in control evolved replicates (σ2 = 0.147).” What makes these results distinct from what is reported on lines 151-152? From my understanding both sentences are reporting on the same finding that Btt replicates evolved in primed hosts show higher variance in virulence than Btt controls when in a primed host. Since there are statistical comparisons being made in the text that are not present in the main figure texts, the authors should have a table reporting their statistical output.
- Figure 2 presentation:
  - Include X axis title for 2A & 2B
  - Y axis should include proportion
  - The authors should present error bars for their boxplots throughout the text.

Figure S1:

- Line 164-What is the authors motivation for including this figure in the supplement? This seems like a significant finding from their evolution experiment (referenced even in the abstract).

“Do the evolved pathogens differ in spore production, sensitivity to priming and fitness?” Section

- Line 167: I believe the headers for these sections could be better served to state the primary finding from the results section. The questions posed in the headers could be better served to open the motivation for the experiments in each section.

Figure 3:

- Line 174: Swap figure order with Figure 3C since Figure 3D is mentioned first.
- Figure 3 presentation:
  - The x and y axis should be a larger font.
  - What is the unit of the Y axis? Is it on a log scale?

Figure 4:

- Line 194: In the legend the authors state what virulence is in this case, but it should be reflected in the text as well or more explicitly say host mortality.
- Line 205: The authors state “found a negative trend for the primed-evolved pathogens” when correlating priming sensitivity with pathogen fitness. What does this suggest?

“Do the evolved pathogens differ in 220 Cry gene expression?” Section

- Lines 224-225: When the authors discuss the statistics for the qPCR analysis, there needs to be a transition sentence between the discussion of the analysis that pools all the evolved lines together (lines 223-224) into the statistic performed for each experimental line relative to the ancestral strain.

“Do the evolved pathogens differ in their genomes and mobilome?” Section

- Figure 5 presentation: Need to indicate in figure legend what the darker and lighter colors mean for the heat map.
- Line 238: The authors mention virulent and non-virulent lines when referencing their genomics sequencing. What do the authors mean by this? In the figure 5 legend, they reference Fig. 2 for the order of virulence by evolved lines seen in the plot. The text of the results section does not formally report which of the evolved strains are the most and least virulent, which should be included.
- Line 271: The relative expression of the *cry3Aa* gene I assume is the qPCR expression measured in the previous section? This should be clarified in the body of the text when presenting Fig. 6B.

Figure S7:

- This supplementary figure needs clear labeling for what each of the plates represent. If possible, zoom in to a portion of the plate so the phage patches are distinct.

**Discussion:**

- Line 348: I do feel like these are reaches in the discussion and takes away focus from the paper. Could be worth future investigation to explore
- Line 357: Is it a fair comparison to make between pseudomonas and bacillus? Gram-negative vs Gram-positive bacteria could very likely evolve different trajectories since they vary greatly
- Line 361: What are the specific pathogen cues triggering immune priming in their study?
- Line 377: While measuring the host’s immune response to evolved pathogens could be a follow-up study, I think it could be a worthy addition to the paper to compare a more virulent evolved line vs a less virulent evolved line to see how they may impact the hosts immune response. Else, I think this point could be further clarified in the discussion of known examples of pathogens that reduce the immune response in beetle larvae.

**Methods:**

- Line 489: How long were these cadavers sitting before being homogenized? For the selection experiment above it says the authors waited 7 days. Is that what is happening here?
- Line 560: What do the authors define as plate in this model?
- Line 581: The authors determined *Btt* fitness in the host by “combining the spore load and proportion of mortality”. How was this performed? Were they summed, made relative to each other? They also mention this in the results section (line 199-200) but do not clarify how they combined these values together.

**Grammar**:

Below are a few examples in the text that could be rephrased for clarity. Working with a copyeditor could help improve the flow of the text.

- Line 141-142: The authors write ‘Regarding the host environment encountered during the readout, i.e. after evolution had happened’. They use ‘i.e.’ frequently throughout the text, so it could help readability to make sentences like this more succinct.
- Line 398: Sentences starts with “As pathogen”, this can be rephrased.
- Line 507: Possible error with citation manager?

**Confidential comments to editor:**

I do not have expertise in conducting genomic sequencing experiments, so I do not have confidence giving feedback on how these data were analyzed and reported.
