# Supplementary material for: Experimental evolution of a pathogen confronted with innate immune memory increases variation in virulence
Source: PLoS Pathog. 2025 Jun 18;21(6):e1012839. doi: 10.1371/journal.ppat.1012839 (PMC12176410; doi:10.1371/journal.ppat.1012839)
Supplement: Attachment — Submitted filename: Answer_revision_21_3.doc [file ppat.1012839.s013.doc]

| University of Münster \| Institute for Evolution and Biodiversity \| Hüfferstraße 1 \| DE 48149 Münster |  |  | Prof. Dr. Joachim Kurtz |
| --- | --- | --- | --- |
|  |  |  |  |
|  |  |  | University of Münster  Institute for Evolution and Biodiversity  Hüfferstraße 1  DE 48149 Münster  Germany  Phone +49 251 83 24661  Fax +49 251 83 24668  joachim.kurtz@uni-muenster.de  30.11.2026 |
|  |  |  |  |

Dear Dr Unckless,

We are pleased to resubmit our manuscript and would like to express our sincere gratitude to the three reviewers as well as you and other editors involved for their thoughtful comments and constructive suggestions. Their valuable feedback has greatly contributed to improving the clarity and quality of our work.

In the attached response, we have addressed all the points raised by the reviewers in detail. We appreciate the time and effort they dedicated to evaluating our manuscript, and we hope that our revisions meet their expectations.

We look forward to your feedback and remain hopeful for a positive outcome.

Sincerely, on behalf of all authors,

Ana Korša and Joachim Kurtz

Additional Editor Comments (if provided):

Three experts in the field have reviewed your manuscript entitled "Experimental evolution of a pathogen confronted with innate immune memory increases variation in virulence". All three were enthusiastic about the work but pointed out significant concerns. All three reviewers point to references in the literature that should be considered in a revision and to a more careful grounding of the current hypotheses in terms of the available literature. From an editorial perspective, my biggest concern was raised by Reviewer #2 who asked whether the increased variance observed was the result of comparing technical to biological replicates in the experimental vs. control populations. If so, can we really say anything about increasing variance in the primed populations? All three reviewers also asked for more clarity in discussing aspects of the results (implications for selection, justification for fitness proxies, discussion of other virulence factors). Finally Reviewer #2 suggests a few additional experiments (pathogen load, pupation rate). I wonder whether you have that pupation rate data and could include it as another measure of fitness.

We sincerely appreciate your positive feedback and the constructive comments provided by the three reviewers. Their insightful suggestions have helped us refine our manuscript, and we have made several key improvements based on their recommendations.

We have incorporated additional literature and revised our theoretical framing in the introduction to reflect a more neutral question, rather than a directional hypothesis.

Regarding the concern about technical vs. biological replicates in the ancestral population, we acknowledge that direct comparisons between the ancestral and evolved populations do not allow conclusions about increasing variance. However, the ancestral population was included purely as a reference to see in which direction virulence evolves, and our conclusions are based on comparisons between the primed evolved and control evolved bacteria. Importantly, both of these consist of 8 biological replicates (independently evolved lines) each, rather than technical replicates. We have incorporated new analysis suggested by Rev1 and now clearly show and state in the text the comparison between primed and control evolved (Fig 2). **Lines 185-201**

In response to the suggestion for additional experiments, we do not have pupation rate data. However, given that *Bacillus thuringiensis* (*Btt*) is an obligate killer (Raymond et al., 2010), we do not expect other disease phenotypes to be relevant for pathogen fitness. Since infection invariably results in host death, mortality remains the most direct and meaningful measure of virulence.

While previous studies (Milutinović, Fritzlar, & Kurtz, 2014; Baur et al., 2024) have shown that infection and immune priming can influence host size and development, these effects are primarily host-centric and do not provide direct insights into pathogen fitness or virulence.

Given the rapid course of infection (24 hours), measuring pathogen load over time was not feasible. However, we assessed final spore load as an indicator of bacterial replication in the host.

Reviewers' Comments:

Reviewer's Responses to Questions

Part I - Summary

**Reviewer #1:** This study uses an experimental evolution approach to test whether innate immune priming selects for higher pathogen virulence, using red flour beetles and their Bacillus pathogen as a model system. Understanding effects of innate immunity on virulence evolution is a very important and relevant question in many systems, and the authors use an ideal model system to test this question. Overall the methods and analysis are well done, and the results are quite interesting in that virulence did not increase, but became more variable after experimental evolution in primed hosts. I think overall that this is an exciting and important contribution to the literature.

We thank the reviewer for their positive feedback.

**Reviewer #2:** The authors examined how immune priming by pathogens influences the evolution of virulence. While virulence did not differ across primed and control host treatments, there was greater variation in terms of virulence across replicates in primed hosts. Although this particular result was interesting, I have several concerns:

**Line 95: The claim that this is the first study to show the influence of immune priming on pathogen evolution is not accurate. Previous studies, which the authors cited, have examined the interaction between live pathogen exposure (Read 2015), antigen exposure (Barclay 2012), and microbiota exposure (Hoang 2024), on the immune system and how they shape pathogen evolution. As immune priming is a general phenomenon, saying that immune priming has not been examined before in the context of pathogen evolution is misleading.**

We have revised the wording to specify 'insect immune priming.' While we acknowledge that immune priming is a broader phenomenon, our study specifically investigates its role in pathogen evolution in insects – specific immune response, which distinguishes it from the cited studies on live pathogen exposure, antigen exposure, and microbiota exposure. We hope this clarification addresses the concern and better highlights our work's novelty in insect immune priming. **Line 132**

Line 360: With regards to specificity: has it been shown that nothing else could have triggered the same response from the beetle host?

We thank the reviewer for requesting more details regarding the important aspect of specificity of the priming response. Indeed, previous work has demonstrated that priming in *T. castaneum* is specific for the bacterial strain, meaning that exposure to priming-inducing culture supernatants derived from *Bacillus thuringiensis tenebrionis (Btt)* trigger a priming response that protects only against this strain, but not other strains of *B. thuringiensis* (Futo et al. 2017). Recent work has confirmed such specificity and identified virulence factors present in the culture supernatants as the most likely priming agents (Länger et al. 2023; Baur et al. 2025), further supporting the specificity of the priming response to *Btt*. We have slightly adjusted the sentence and added relevant citations in the manuscript to make this clearer (l. …). **Line 406-407**

The significant finding of this study, where variation increased across replicate populations, is interpreted as “evolutionary relevant because selection needs variation to act upon” (line 388). This interpretation is incorrect has evolution acts on the variation within a population, not across independently evolving populations.

We understand the reviewer's point that evolution acts on variation within a population rather than across independently evolving populations. However, in natural systems, pathogens can evolve in separate host populations that may remain isolated for some time but eventually come into contact. This dynamic is well captured by the concept of metapopulations, where local populations experience independent evolutionary trajectories but can exchange individuals or genetic material through migration or other interactions. For example, in the case of flour beetles in different silos, pathogen populations may evolve separately and later mix, allowing selection to act on the variation that has accumulated across these populations. In this context, the observed increase in variation across replicate populations could still be evolutionarily relevant. We added a sentence to the manuscript to make our point clearer. **Line 446-450**

Line 164: **The increase in variation from the ancestor is due to the ancestor having technical replicates instead of biological replicates? So an increase in variation would be expected.**

We thank the reviewer for their comment! It is certainly correct that the ancestor is represented by technical replicates, but it is important to note that our main comparison is between biological replicates (lines) that independently evolved in either non-primed (control) or primed hosts, i.e., both consisting of biological replicates. To make this clearer, and to also incorporate a comment of Rev. #2, we performed additional analyses to capture the variation. Ancestor here serves purely as a reference control to see in which direction virulence evolves, but it is not our main question. The results support that the variation is greater in primed-evolved compared to control-evolved pathogens (Fig2). We have also changed the wording to make this clearer **Line 195-201**

Lines 366 – 367: the way this is worded suggests that immune priming has been shown to maintain microbial variation through balancing or pleiotropic effects, when neither citation has to do with immune priming.

We thank the reviewer for pointing this out. Our intention was not to suggest that immune priming itself maintains microbial variation through balancing or pleiotropic effects. Rather, we meant that immune priming could lead to similar conditions. However, as this might be misleading, we have removed these citations and revised this part to more accurately reflect our intended meaning. **Line 412-417**

The Discussion was a bit too long in general.

We appreciate the comment. We shortened the discussion in some places. However, other reviewers suggested to expand on certain things.

**Reviewer #3:** In this study, Korša et al asked whether immune priming in the insect host Tribolium castaneum result in the evolution of increased virulence in the entomopathogenic bacteria Bacillus thuringiensis tenebrionis (Btt). Using a one-sided experimental evolution set-up, they subject Btt to undergo selection pressure in either an immune primed host or non-primed host for 8 selection cycles. To test the effects of their selection treatments, they performed a common garden experiment where evolved-lines of Btt and the ancestral strain were phenotyped for host-mortality (virulence) and spore load (transmission) in either an immune primed host or non-primed host environment. The authors observed that both evolved Btt lines did not increase virulence or transmission, in-fact they were reduced, when infecting primed or non-primed hosts. Interestingly, they found that the selection pressure of immune priming resulted in increased variation in Btt virulence. Genomic sequencing of these evolved strains revealed, when compared to the ancestral strain, minimal chromosomal mutations and reduced covered of the plasmid 188 kb, known to carry genes encoding virulence factors like the Cry toxin. The authors find some evidence that the evolved Btt lines have increased lytic phage activity, which could potentially explain reduced sporulation in those lines. While some additional experiments can help complete the presented work, this is an interesting study that interrogates whether incomplete immunity like immune priming can act as a selection pressure for bacteria pathogens to evolve virulence mechanisms against. The study appears mostly sound; however, the authors should revise the language to improve readability.

We thank the reviewer for their positive feedback and provide details regarding the specific suggestions below. We have improved the language in several places of the manuscript.

Part II – Major Issues: Key Experiments Required for Acceptance

**Reviewer #1:** My major concerns are all regarding presentation / interpretation. The main result is higher variation in virulence in pathogen lines evolved in primed hosts. This is quite interesting and I think can be highlighted even more strongly. I also would suggest using something other than variance as your metric presented in the results-I would encourage the authors to look at this paper which has been very helpful to my own lab group in thinking about how best to quantify differences in variability between groups: https://besjournals.onlinelibrary.wiley.com/doi/full/10.1111/2041-210X.14197

We much appreciate the suggestion of Reviewer 1 and added the estimation of the coefficient of variation by estimating it from our posterior predictions. Using this approach allows for a distribution of K-CV values that can be compared, and changes in variance can be calculated as log2 fold change. K-CV is a correction of the traditional Pearson coefficient of variation. By standardizing the p-cv, this metric can be safely applied to proportional data and provides an estimation of the relative degree of covariation on a scale of 0 to 1. The Figure 2 has been updated. We have now added the analysis and showing the results in Figure 2 and results section is updated. **Line 195-201**

In terms of interpreting the variance result, in the discussion there is very little time spent on the implications of this for selection. At the very end, you note that such variation may boost evolutionary potential (line 389), yet you also saw no signal of directional selection because of this variability, which you note may reflect different evolutionary routes taken by each line. I think these implications are worth more focus in the discussion- do you think that evolutionary potential truly is higher in the primed treatments, and you just needed more generations of selection to see any signals of evolution? Overall I would have liked to see more extended discussion of the implications of what, to me, was a really interesting and exciting result (higher variation in bacterial lines evolved in primed hosts). This seems really novel and exciting, and broadly parallels some results we are finding in bacterial gene expression in songbirds with and without prior pathogen exposure (still as yet unpublished).

We are thankful for this insightful comment. We have now extended our discussion on both, potential reasons for the observed increased variance and its implications **Line 409-421;424-428;447-450**

We agree that the implications of increased variance in bacterial virulence in the primed-evolved lines warrant further discussion, particularly regarding the role of selection and evolutionary potential.

In general, changing environments tend to favour increased phenotypic and genetic variation, as variation is essential for adaptation to fluctuating conditions. In our study, the primed host environment may have acted as a more dynamic selective landscape, maintaining higher variation in bacterial traits. This aligns with theoretical expectations that variable environments select for broader evolutionary potential by preserving diverse phenotypes that could be advantageous under future conditions.

While we observed higher variation in virulence within the primed-evolved lines, we did not detect a clear signal of directional selection toward increased virulence. One possible explanation is that the evolutionary trajectories of each replicate line were divergent, meaning different adaptive strategies may have emerged rather than a consistent trend toward higher virulence. Alternatively, the increased variance itself may be an early-stage evolutionary response, with directional selection requiring more generations to become apparent. It is possible that given a longer experimental timeline, we would begin to see a clearer pattern of adaptation, potentially in the direction of increased virulence

In several places (including Line 387) you conclude that you show an effect of specific immune priming on the evolution of bacterial virulence. Can you really state that given your results that virulence did not change, other than decreasing in both selected lines relative to the ancestral state? I think rewording may be needed.

We thank the reviewer for the comment. We recognize that our original phrasing may have overstated the effect of specific immune priming on the evolution of bacterial virulence. We have revised the wording to more accurately reflect our findings, emphasizing how the bacterial pathogen evolves in response to specific immune priming of the host. **Line 445-446**

Finally, this is more minor, but I think more justification for your proxy for “fitness” is needed- you simply note that you combine mortality rates and spore load data (Lines 199-200) but even in the methods, there is no justification for why this is a reasonable proxy for bacterial fitness in this system.

We appreciate the suggestion. We agree that more justification was needed for our proxy for bacterial fitness. We have now clarified in the manuscript that this approach was chosen because it captures both key components of *Btt’s* transmission strategy: host mortality, which is necessary for transmission as *Btt* is an obligate killer, and spore load, which reflects the pathogen’s replication within the host and its potential to spread. This explanation has been added to the manuscript accordingly. **Line 560-562**

Reviewer #2: (No Response)

Reviewer #3: 1. What prior literature are the authors using to base their hypothesis that immune priming would evolve for higher virulence in Btt? They cite Hoang, et al (2024) and Rafaluk et al (2017) as examples of evolved higher virulence, but they also cite evidence from Ford, et al (2016) that pathogens evolve lower virulence. Mikonranta et al (2015, BMC Ecology and Evolution, https://doi.org/10.1186/s12862-015-0447-5) show that one-side evolution of Serratia marcescens DB11 in D. melanogaster resulted in reduced virulence, which the authors did not cite. It would be helpful to clarify what aspects from the literature motivate the authors hypothesis that Btt evolves higher virulence in response to immune priming.

We thank the reviewer for their thoughtful feedback. Indeed, our formulation was not very clear and therefore we have adjusted our wording that implied we had a directional hypothesis **(24-25; 89-93; 106-107).** We would like to clarify that we did not expect that immune priming necessarily favours the evolution of higher virulence. Rather, as we state in our introduction, the existing literature provides examples where incomplete host immunity has influenced pathogen virulence in different directions, either increasing or decreasing it. These contrasting outcomes highlight the need for more empirical studies to understand the conditions under which incomplete immunity selects for higher or lower virulence. Our study is, therefore, exploratory in nature, aiming to examine the evolutionary consequences of immune priming in this system without presupposing a specific directional outcome. We acknowledge the relevance of Mikonranta et al. (2015) and appreciate the suggestion to include this reference, which now is cited in the discussion (**354)**

2. In the second paragraph of the discussion section, the authors discuss three possible explanations to justify the observed lower virulence of evolved Btt lines: 1) evolution of high virulence is costly in the absence of co-evolution, 2) virulence factors encoded on mobile elements like plasmids are costly, 3) virulence evolution could be reduced due to other microbes in the host environment. While the authors do a great job discussing point two, the description of points one and three are minimal and could be expanded on what aspects of their study correspond with these points they cite? Additionally, in point 2, the authors discuss Cry toxins do not play a critical role for Btt virulence in beetles, so why focus on measuring Cry3Aa expression over other virulence factors? Were there any mutations observed in virulence factors like chitinases or proteases, or does plasmid 188kb encode additional virulence genes? Using the existing sequencing results, I think the current study would benefit from the addition of reporting of how other virulence factors were impacted in the evolved lines.

We thank the reviewer for their detailed feedback. We appreciate the suggestion and we have changed the discussion a bit, mentioning how our study as well was one sided adaptation of the pathogen. **Line 352-354**

Point 2: Regarding the role of Cry toxins, we are sorry if this might have been misunderstood. Cry toxins are among the most relevant virulence factors in *Btt* for *Tribolium castaneum,* as they play a critical role in pathogenicity. We don´t think this is not a relevant factor of toxicity, but it was shown that the *Cry* toxin alone is not sufficient to kill the larvae, rather the combination of spores and toxins (which is also the natural way of infection). We focused on measuring cry3Aa expression because it is a well-characterized insecticidal toxin in *Btt* with known effects on beetle hosts. However, we agree that other virulence factors, such as chitinases and proteases, could also contribute to pathogenicity and may evolve in response to host immune priming. However, we did not detect any notable mutations in known virulence factors, suggesting that changes in virulence may be driven by regulatory or expression-level differences rather than genomic alterations.

Point 3. We agree that our discussion of this point was minimal and we expanded a bit. **Line 372-378**

3. The potential increase in lytic phage activity in both evolved lines in Btt is interesting and I think could be expanded on in this study. In the double layer agar experiments, the ancestral strain acts as a negative control since it does not have active phages, so repeating this experiment and the other phenotypes measured (mortality and sporulation) with a Btt strain known to have active phages can be a positive control. Thus, if a Btt line with active phages show similar phenotypes to the evolved strains, potentially, this increased lytic activity maybe a mechanism for reduced virulence. Also, is there a difference in the number of phages between immune primed and non-immune primed evolved strains? The double-layer agar assay is quantifiable, so it should be feasible to score the number of phages to test whether the primed and non-primed evolved strains differ.

We appreciate reviewer´s suggestions on expanding this aspect of our study and testing whether increased lytic activity could be a mechanism for reduced virulence. However, at the moment we are not able to expand on this study.

Regarding the use of a *Btt* strain with active phages as a positive control, to our knowledge, no such strain is currently available for use in this context. Because of this, our approach has relied on comparing the evolved strains to the ancestral which does not have active phages, as a negative control. While a positive control would be valuable for further validating our findings, the absence of a known *Btt* strain with active phages prevents us from including this in our current experimental framework.

With respect to quantifying phage abundance in immune-primed versus non-primed evolved strains, our genomic analyses did not reveal substantial differences between these two treatments. While phage activity seems to differ between ancestral and evolved populations, it does not appear to be a dominant driver of divergence between control- and primed-evolved lines (i.e., the more interesting comparison). While generally possible, quantifying phage numbers using the double-layer agar assay is not as easy as it seems, because our preliminary studies have indicated that results may vary largely due to slight variations in bacterial culture conditions. However, we believe our genomic data already provide key insights into phage dynamics across the evolved strains, supporting our interpretation of their potential role in virulence evolution.

4. One of the major findings in this paper is that evolution to immune priming results in increased variation to virulence, however, the authors only show host mortality as the only phenotype to demonstrate this. Increased host mortality is associated with higher virulence phenotypes in pathogens. Since the authors found that Btt evolved in beetle larvae exhibit reduced virulence, I think the study could be complemented by including at least one or two other disease phenotypes associated with virulence. For example, measuring Btt pathogen load during the course of infection or measure pupation rate of larvae that survive acute infection.

We thank the reviewer for this point! We understand the point and we were also considering measuring other phenotypes. However, as *Btt* is an obligate killer (Raymond et al. 2010), we would not expect other disease phenotypes to be relevant for the pathogen. The nature of this system means that infection invariably leads to host death, making mortality the most direct and meaningful measure of virulence. While it is known that infection and immune priming can influence host size and development (Milutinović, Fritzlar, and Kurtz 2014; Baur et al. 2024), these effects are primarily host-centric and do not provide significant insights into pathogen fitness or virulence. Additionally, given the rapid course of infection (24 h), measuring pathogen load over time was not feasible, though we did assess final spore load as an indicator of bacterial replication in the host.

Part III – Minor Issues: Editorial and Data Presentation Modifications

Reviewer #1: Line 151 – Is variance really the best measure here? See major comments.

We have addressed this point in the major comments.

Line 159 – can you justify why you used a beta distribution here, versus something like a gamma distribution. I didn’t see justification in the methods either.

We thank you for pointing this out and we added more explanation in the method part. As survival is measured as the proportion of surviving individuals, it is bounded between 0 and 1. Traditional models, however, often assume constant variance in survival models, which may not hold when survival rates vary across groups. The Beta distribution allows for flexible modelling of survival probabilities therefore capturing different survival patterns while explicitly accounting for heteroscedasticity. Its variance depends on both the mean survival proportion (μ) and the precision parameter (ϕ), ensuring a more accurate representation of uncertainty in survival outcomes. Hence, the variance of the response variable in a beta regression is not constant but depends on both the mean survival proportion (μ) and a precision parameter (ϕ) can be calculated as Var(Y)=μ(1−μ)/ 1+ϕn. **Line 638-647**

Line 173- I suggest deleting the word “where”

We changed that

Lines 391-393- The sentence “Not only the medical relevance…” is not grammatically complete. We changed it.

Line 507- there is an error here in the reference source that needs updating.

We changed that

Reviewer #2: Methods and Figures

Line 152: why is Fig. 2A presented after 2B?

We have a different figure now and we corrected the order.

Line 198: the p-value isn’t significant here? The significance threshold was not discussed in the Methods.

We thank the reviewer for noticing this. It was a mistake in wording. We changed it. **Line 233-234**

Lines 233: Was sequencing done at the population level instead of at the clonal? E.g., the heat map of Fig. 5 suggests allele frequencies within populations. What does this mean in terms of connection to the phenotypic results?

We thank the reviewer for pointing this out. Yes, sequencing was performed at the population level rather than at the clonal level. For each evolutionary line, we inoculated 10 independent overnight cultures from single colonies, extracted DNA from these cultures, and pooled the samples before sequencing. This approach allowed us to capture the genetic diversity within each evolved population rather than focusing on individual clones. We added explanation to the method part **(584-587)**

The heatmap in Fig. 5, which shows allele frequencies, reflects this population-level sequencing strategy. The allele frequency data indicate the presence of polymorphisms within populations, rather than fixed mutations in single clones. We applied a fraction threshold of 0.1 (10% read coverage for an alternative allele) to identify relevant variants.

Regarding phenotypic correlations, because we did not use the same clones for sequencing and phenotypic assays, direct genotype-phenotype correlations were not possible. However, population-level sequencing still provides insights into the prevalent genetic variants within each line and their potential contribution to observed phenotypic trends.

Lines 235 – 236: 32 unique variants?

Yes, that is correct, thank you for pointing this out, we changed the text accordingly

Line 239: “position 2,017,894” isn’t really general wording. It doesn’t make sense without reading the Methods first.

We appreciate the comment. For clarity, we changed the nomenclature of the variants according to <https://atlasgeneticsoncology.org/teaching/30067/nomenclature-for-the-description-of-mutations-and-other-sequence-variations>

**Line 279-275**

The data points for Figs. 6a and 7b are not present like they are in previous figures.

We are aware that Figures 6a and 7b differ in presentation compared to the previous figures. Including individual data points, as in the earlier figures, would have made these figures less readable. However, we have adjusted the figures slightly to better illustrate the variation among individual replicates.

Figure 6b: what is the p-value for? what test?

We are thankful for pointing out the missing information regarding the statistical analysis! We mention the model in the figure caption now.

Other comments:

Lines 54 – 55: It is unclear why high virulence would be favored in non-tolerant hosts.

We are aware we didn’t make this clear in the first place. As host population becomes more tolerant, pathogens can enhance their transmission without necessarily increasing host mortality, which allows for the development of greater virulence. However, when these highly virulent pathogens infect intolerant hosts, they can trigger severe illnesses and potentially devastating outbreaks. This can occur because non-tolerant hosts are unable to mitigate the harm caused by high pathogen loads, resulting in increased death rates. Consequently, high virulence is not directly favored in non-tolerant hosts but instead emerges as a result of pathogen adaptation within tolerant populations.

We added more explanation on that. **Line 73-76**

Line 63: what is external host immunity?

Here we mean chemical defence systems comprised of potent antimicrobial compounds released by prospective hosts into the environment - antimicrobial secretions usually produced by insects - a defensive barrier that kills the parasites before the infection occurs (Rafaluk et al. 2017).

We added description in the text- **Line 85**

Line 65: are they contradictory results? They are just examples of ways that virulence evolutionary trajectories can be altered.

We have changed this accordingly **Line 89-90**

Line 67: Incomplete immunity is not defined

We changed the wording to host immunity as we give different examples of how different immune responses influence pathogen virulence and evolution. **Line 89**

Line 90: I’m guessing this is host and not bacteria, in which case hosts are not evolving so they can’t have generations?

We changed this

Lines 141 – 144: how is this different than the next paragraph?

We thank the reviewer for this comment and understand that was a bit confusing. We changed this part of the analysis. **176-195**

Lines 221 – 222: Has cry3a been previously shown to affect virulence of Bt toward the beetle host? Or is this a hypothesis that cry3a is involved and has not been shown before?

The Cry3A toxin has been identified as the specific virulence factor responsible for causing mortality in beetle hosts. Unlike other Bt strains with different Cry toxins, Cry3A demonstrates this outcome(Milutinović et al. 2013; Futo et al. 2017) and might be even responsible for triggering priming response of the beetle (Länger et al. 2023) We have incorporated additional prior research into the text.

Lines 316 – 317: Just a suggestion but it might be better to introduce the coverage data first showing lower cry3aa gene coverage, then go in and test for expression of this gene, and correlation between expression and coverage.

We thank the reviewer for the suggestion. However, we have already tested the expression as one of our phenotypic readouts and prefer to keep this separate from the genomic analysis. The correlation between expression and coverage essentially serves to confirm our qPCR results

Lines 323 – 325: then why did the authors test this gene?

We are sorry that we might have caused confusion regarding the role of the Cry toxin for infecting T. castaneum, and now more clearly describe its role and thus our rationale for testing this toxin (l. **258-259)**. Cry is a major virulence factor of *Btt,* can be costly and some bacteria might lose it. That’s why we first thought this toxin can be responsible for low virulence in some replicates. Our aim was to confirm that the qPCR results aligned with the gene coverage, ensuring we collected RNA samples at the appropriate time point. A lack of difference in expression could potentially indicate that we extracted RNA when the gene was no longer being expressed.

Lines 327 – 330: it is unclear what the logic is here

Thank you for pointing this out. We have slightly changed the sentence – If Cry toxins primarily trigger the priming response, they may not serve as the main drivers of virulence on their own. Instead, their effects might be synergistically enhanced by other virulence factors, such as chitinases, metalloproteases, or vegetative insecticidal proteins. **Line 365-371**

Line 331: do beetles have defensive microbes?

We don’t know about defensive microbes, but we know that microbiota composition changes upon priming treatment (Korša et al. 2022) and that priming response doesn’t happen if the beetles are deprived from their microbiome (Futo, Armitage, and Kurtz 2016). These findings suggest the possibility that certain microbiota members play a role in assisting the host to generate an appropriate immune response or potentially engage in competition with pathogens. We have added more information about this to the manuscript (**l. 372-378**)

Line 372: what specifically about development?

We thank the reviewer for pointing this out. With having more thought about this and other comments in mind, we removed the sentence as this study focuses only on virulence in terms of host mortality as Btt is an obligate killer and any other virulence measures wouldn’t be important for the pathogen.

Lines 392 – 393: how is immune priming being used in these fields?

We thank the reviewer for the comment.

For example, exposure to non-lethal doses of pathogens or immune-stimulating can enhance resistance to subsequent infections in shrimp aquaculture https://onlinelibrary.wiley.com/doi/10.1111/raq.12906

In insect mass production by pre-exposing insects to mild immune challenges, producers may be able to enhance their survival and resistance to microbial infections, ultimately improving production efficiency and product quality.

We incorporate a brief mention of these applications to provide a broader perspective on the practical implications of immune priming**. 453-456**

Lines 507 – 508: error

Thanks for finding this. It is now fixed

Reviewer #3: Here, I list comments specific to each section of the manuscript.

Introduction:

**• More detail on what the authors define as varying levels of virulence would provide welcome context here. For example, line 42 discusses intermediate levels of virulence of the Myxoma virus without describing exactly what intermediate virulence looks like in contrast to high. This could also be revisited in the discussion since the authors did not find evidence for immune primed evolved lines to increase virulence, potentially Btt evolved intermediate virulence mechanisms.**

We thank the reviewer for this suggestion. We agree that providing more detail on how we define varying levels of virulence, including what constitutes "intermediate" virulence, would strengthen the clarity of our discussion.

When we refer to Myxoma virus evolving intermediate virulence, we mean that it has reached a level of pathogenicity where it balances host mortality and transmission efficiency. In contrast to highly virulent strains that rapidly kill hosts—potentially reducing transmission opportunities—intermediate virulence allows for sustained infection and transmission while still overcoming host defenses. We took the comment into account and added this in **Line 60-62**

In the discussion, we acknowledge that we did not observe an increase in virulence compared to ancestral in immune-primed evolved lines **(350-354)** However, our main comparison is virulence between primed and control evolved lines and there, we see that primed evolved lines have a higher probability of higher virulence than control evolved line (Fig 2).

• More information on the activated immune response of primed Tenebrio castaneum vs an active immune response in infected, non-primed larvae would be additional context. For example, description of what are the primary effectors (e.g. antimicrobial peptides, ROS, immune cells) of T. castaneum that are upregulated after infection and priming and which aspect of the response primarily control Btt infection. Additionally, more information on the Btt infection cycle in larvae in the wild would-be helpful context such as including how larvae would encounter spores in nature and how spores reproduce and disperse from larvae cadavers.

We are thankful for the feedback! We have included a more detailed description of the key immune effectors activated in *T. castaneum* following infection and priming. Specifically, we highlight that antimicrobial peptide (AMP) genes and genes involved in cellular immune responses are upregulated only upon Btt infection, regardless of priming status. In contrast, immune recognition genes and reactive oxygen species (ROS)-related genes are upregulated upon priming with *Btt* (but not control) even without subsequent infection. This suggests that priming induces a heightened immune "alertness," potentially providing a more rapid or effective response upon secondary exposure. We added additional description **Line 102-107**

Additionally, we have expanded our introduction on the natural infection cycle of *Btt* in the host. **Line 109-118**

• The authors briefly discuss how external host immunity (line 63) and defensive microbes (line 64) are examples of factors that can impact pathogen virulence without much detail on how they define these terms. For clarity, it would be helpful to provide additional context on the type of pathogen and the infecting host from these cited studies is helpful to know because unique properties of different pathogen species and their distinct host environment could influence what virulence strategies they evolve.

We thank reviewer for this valuable suggestion. We agree that providing additional context on the type of pathogen and host involved in the cited studies will help clarify how external host immunity and defensive microbes influence pathogen virulence and added relevant information (**Line 86-89).**

• Do the authors consider transmission as a form of virulence? I believe they are connected, but the authors should clarify what makes virulence and transmission distinct.

We thank the reviewer for pointing this out. Since virulence is measured as the damage inflicted on the host, mortality is the most relevant measure of virulence in our study, as *Btt* is an obligate killer. In this system, pathogen transmission occurs only after host death, meaning that transmission is better interpreted as a measure of pathogen fitness—specifically, how many spores *Btt* successfully produces within the host. We have added the description of the measure of fitness in the method part **560-562**

Results:

“Does host immune priming affect pathogen virulence evolution?” Section

Figure 1:

• What is the motivation or prior work used to justify performing 8 selection cycles, and how many generations of Btt (doubling time) occur during infection? The papers they cite like Hoang, et al (2024) performed 14 selection passages on P. aeruginosa and Ford, et al (2016) passaged Enterococcus faecalis and Staphylococcus aureus for 10 passages.

We thank the reviewer for pointing this out. Prior work has shown that *Btt* undergoes approximately 9.5 generations in the host (Milutinović et al. 2015), so we estimate that around 76 generations in the host occurred in our experiment. We have added this to Lines **141-142** Furthermore, we decided to stop at generation 8, as some of our replicates were not inducing significant mortality in the host, and we were concerned about losing these populations. The papers we cite performed passaging in C. elegans, where it is easier to maintain the host, whereas in Tribolium, producing new larvae takes about 14-15 days. To maximize statistical power, we chose to conduct the experiment in 2 blocks with more replicates, rather than increasing the number of selection cycles at the expense of fewer replicates.

• For clarity, I think it is helpful to have a prescribed naming convention for the immune primed evolved Btt and the non-primed (author refers to control) evolved Btt to be distinct from the immune primed host environment and the control host environment.

We thank reviewer for their comment. We understand the importance of a clear naming convention to distinguish between the evolved *Btt* lines and the host environments. After careful consideration and discussions with colleagues, we decided to maintain the terminology "primed-evolved" and "control-evolved" for the bacterial lines, as these terms directly correspond to the experimental regimes under which they evolved. Similarly, we use "primed host environment" and "control host environment" to describe the conditions in which the bacteria were exposed during evolution. We initially also considered but then decided against using a different term for the evolution condition (‘non-primed’) and the common-garden environment (‘control’), because the host conditions are actually the same in both, and this might get lost when using different terms. We believe this terminology maintains consistency while accurately reflecting the experimental design.

Figure 2:

• Line 142-143: Here the authors are trying to directly compare average mortality between control hosts and primed hosts infected with ancestral, immune primed, and non-immune primed Btt. I think the data across these two figures should be compiled in the same graph if they are going to make direct make the comparisons between the two host environments.

We thank the reviewer for this suggestion, but please note that Fig. 2A compiles the data into one graph showing the average mortality of each replicate line in both the primed and control host environment, showing how all replicates induce less mortality in primed hosts, allowing for a direct comparison of how each line performed in the two host environments. Additionally, the figure has been changed because of the new analysis suggested by Rev1. To asses the variance we performed a Bayesian model in primed and control host environment separately and we would like to separate the figures accordingly.

• Line 145: What makes this analysis different than the one performed above? I'd explicitly state what type of model you created in the body of the results and what are the major factors being tested.

We thank the reviewer for this point and we have added the model into the body of the text. We have moved the other analysis as it was redundant and we changed the analysis as suggested by Rev1

• Line 156: The authors state “the primed host, we observed higher variance in virulence across primed-evolved replicates (σ2 = 0.862) than in control evolved replicates (σ2 = 0.147).” What makes these results distinct from what is reported on lines 151-152? From my understanding both sentences are reporting on the same finding that Btt replicates evolved in primed hosts show higher variance in virulence than Btt controls when in a primed host. Since there are statistical comparisons being made in the text that are not present in the main figure texts, the authors should have a table reporting their statistical output.

We are grateful for the comment as we have also been struggling what is the best way to show and report this. However, we have removed this part as we performed another analysis.

• Figure 2 presentation:

o Include X axis title for 2A & 2B

Thank you for the comment. However, we think adding the x titles would make figure more ‘busy’ and in the newly arranged figure, the selection treatments are now stated prominently in the legend of Fig. 2A close to Fig. B-E.

o Y axis should include proportion

We have added this

o The authors should present error bars for their boxplots throughout the text.

We appreciate the suggestions. However, as the plots are showing the mean and 95%CI, standard error bars seem inappropriate. Note also that we changed some of the plots.

Figure S1:

• Line 164-What is the authors motivation for including this figure in the supplement? This seems like a significant finding from their evolution experiment (referenced even in the abstract).

We are thankful for this suggestion. We agree and have now changed the analysis as suggested by Rev1 and included this as Fig. 2 D, E into the main part with slightly changed representation as suggested.

“Do the evolved pathogens differ in spore production, sensitivity to priming and fitness?” Section

• Line 167: I believe the headers for these sections could be better served to state the primary finding from the results section. The questions posed in the headers could be better served to open the motivation for the experiments in each section.

Thank you for your comment, and that is indeed a good point. We also had detailed discussions about this. However, we believe that framing the section with a question is better suited for the results part, as the chapter itself provides the answer to that question. Often, these answers are more complex than a simple statement as header could grasp. For example, the first result chapter shows that the mean virulence did not differ between evolution treatments, while the variance did. A statement in the header could only highlight one of these findings.

Figure 3:

• Line 174: Swap figure order with Figure 3C since Figure 3D is mentioned first.

We have changed that accordingly

• Figure 3 presentation:

o The x and y axis should be a larger font.

We have enlarged the font.

o What is the unit of the Y axis? Is it on a log scale?

This is a linear scale. And its number of spores per mL. We adjusted this.

Figure 4:

• Line 194: In the legend the authors state what virulence is in this case, but it should be reflected in the text as well or more explicitly say host mortality.

We are thankful for the comment. We wanted to focus on virulence as this is the property of the pathogen measured in the host and since our focus is on the pathogen, not the host, we want to emphasize virulence.

• Line 205: The authors state “found a negative trend for the primed-evolved pathogens” when correlating priming sensitivity with pathogen fitness. What does this suggest?

The negative trend indicates that as the fitness of the primed-evolved pathogens increases, their sensitivity to host immune priming decreases. In other words, more fit pathogens appear to be less impacted by the host's primed immune response. This suggests that these higher-fitness strains may have evolved mechanisms to better resist or evade the immunological effects associated with priming **Line 242-243**

“Do the evolved pathogens differ in 220 Cry gene expression?” Section

• Lines 224-225: When the authors discuss the statistics for the qPCR analysis, there needs to be a transition sentence between the discussion of the analysis that pools all the evolved lines together (lines 223-224) into the statistic performed for each experimental line relative to the ancestral strain.

Thank you for this suggestion; we added such a sentence (262-264)

“Do the evolved pathogens differ in their genomes and mobilome?” Section

• Figure 5 presentation: Need to indicate in figure legend what the darker and lighter colors mean for the heat map.

Very good point. We updated the figure captions of figures 5 and 7 to better highlight the actual representation within the plots

• Line 238: The authors mention virulent and non-virulent lines when referencing their genomics sequencing. What do the authors mean by this? In the figure 5 legend, they reference Fig. 2 for the order of virulence by evolved lines seen in the plot. The text of the results section does not formally report which of the evolved strains are the most and least virulent, which should be included.

We thank the reviewer for pointing this out. We changed the wording regarding virulent and non-virulent strains. However, in Fig 5 we wanted to see if there is pattern with virulence so we adjusted the lines according to their virulence which is indicated in Fig 2 from left to right.

• Line 271: The relative expression of the cry3Aa gene I assume is the qPCR expression measured in the previous section? This should be clarified in the body of the text when presenting Fig. 6B.

We appreciate the suggestion and have added additional explanation. **Line 311-312**

Figure S7:

• This supplementary figure needs clear labeling for what each of the plates represent. If possible, zoom in to a portion of the plate so the phage patches are distinct.

We are thankful for the suggestions! We worked them in and hope the updated figure presents itself in a better way

Discussion:

• Line 348: I do feel like these are reaches in the discussion and takes away focus from the paper. Could be worth future investigation to explore

We appreciated the comment, and we have removed the sentence.

• Line 357: Is it a fair comparison to make between pseudomonas and bacillus? Gram-negative vs Gram-positive bacteria could very likely evolve different trajectories since they vary greatly

We thank the reviewer for the comment. We acknowledge that different pathogens may follow different adaptive trajectories at the molecular level. It is worth noting, however, that in this case, the priming or partial host immunity was non-specific and triggered by the host's microbiota, while in our study priming is specific for the bacterial strain and triggered by pathogen-derived cues present in the supernatants used for priming. Finally, different host-pathogen systems may show divergent evolutionary trajectories. We have added the explanation in the discussion. **Line 402-408**

• Line 361: What are the specific pathogen cues triggering immune priming in their study?

We thank the reviewer for the comment. The specific pathogen cues that trigger immune priming are not fully clear yet, but we have recent evidence that virulence factors, including the Cry toxin, play a role. We previously performed proteomic analyses comparing priming-inducing filter-sterilized supernatant from Btt with a bacterial strain that does not induce priming and observed differences in the substances present (Länger et al. 2023; Baur et al. 2024).

• Line 377: While measuring the host’s immune response to evolved pathogens could be a follow-up study, I think it could be a worthy addition to the paper to compare a more virulent evolved line vs a less virulent evolved line to see how they may impact the hosts immune response. Else, I think this point could be further clarified in the discussion of known examples of pathogens that reduce the immune response in beetle larvae. **Line 432-438**

We are thankful for this thoughtful suggestion. While examining host immune responses to evolved pathogens would indeed provide valuable insights, our current study focuses on pathogen evolution rather than host immunity. Additionally, due to time constraints, we are unable to incorporate these additional measurements in this study. However, we recognize the importance of this aspect and will further clarify the discussion by elaborating more:

It has been previously shown that Bacillus thuringiensis supresses the immune system of diamondback moth. Furthermore, it has been previously shown *that Tribolium castaneum* exhibits a distinct immune response upon oral infection with *Bacillus thuringiensis*. Specifically, genes associated with the Toll and Imd pathways were downregulated following oral infection (Behrens et al. 2014). Future research could focus on investigating specific immune markers in the host, such as phenoloxidase activity, antimicrobial peptide expression, or hemocyte counts, to determine whether evolved pathogens actively suppress immune responses. This would help clarify whether the observed fitness differences among strains result from enhanced immune evasion mechanisms.

Methods:

• Line 489: How long were these cadavers sitting before being homogenized? For the selection experiment above it says the authors waited 7 days. Is that what is happening here?

Yes, we also waited 7 days. We clarified this in the method part.

• Line 560: What do the authors define as plate in this model?

Plate is a random factor as beetle larvae were put on infection diet in the 96 well plate. Each plate contained spores from all pathogen lines. This is described in detail in the methods part.

• Line 581: The authors determined Btt fitness in the host by “combining the spore load and proportion of mortality”. How was this performed? Were they summed, made relative to each other? They also mention this in the results section (line 199-200) but do not clarify how they combined these values together.

We thank the reviewer for the comment. We calculated Btt fitness by multiplying the spore load with the proportion of host mortality. Since Btt is an obligate killer, its fitness depends on both the number of hosts it successfully infects and the spores produced per infected host. We have clarified this methodology in the manuscript to ensure transparency **Line 560-562**

Grammar:

Below are a few examples in the text that could be rephrased for clarity. Working with a copyeditor could help improve the flow of the text.

• Line 141-142: The authors write ‘Regarding the host environment encountered during the readout, i.e. after evolution had happened’. They use ‘i.e.’ frequently throughout the text, so it could help readability to make sentences like this more succinct.

• Line 398: Sentences starts with “As pathogen”, this can be rephrased.

• Line 507: Possible error with citation manager?

We sincerely appreciate the reviewer’s comments regarding language clarity and readability. As non-native English speakers, we acknowledge that our writing may not always be as fluid as desired. However, we have made efforts to improve the manuscript using available language tools.

In response to the specific points raised:

We have revised sentences that frequently use i.e. to enhance readability and ensure smoother flow.

The sentence beginning with “As pathogen” (Line 398) has been reworded for improved clarity.

We have also reviewed and corrected any citation formatting issues, including the one on Line 507.

PLOS authors have the option to publish the peer review history of their article (what does this mean?). If published, this will include your full peer review and any attached files.

If you choose “no”, your identity will remain anonymous but your review may still be made public.

Do you want your identity to be public for this peer review? For information about this choice, including consent withdrawal, please see our Privacy Policy.

Reviewer #1: No

Reviewer #2: No

Reviewer #3: No

[NOTE: If reviewer comments were submitted as an attachment file, they will be attached to this email and accessible via the submission site. Please log into your account, locate the manuscript record, and check for the action link "View Attachments". If this link does not appear, there are no attachment files.]

Figure resubmission:

While revising your submission, please upload your figure files to the Preflight Analysis and Conversion Engine (PACE) digital diagnostic tool, https://pacev2.apexcovantage.com/. PACE helps ensure that figures meet PLOS requirements. To use PACE, you must first register as a user. Registration is free. Then, login and navigate to the UPLOAD tab, where you will find detailed instructions on how to use the tool. If you encounter any issues or have any questions when using PACE, please email PLOS at figures@plos.org. Please note that Supporting Information files do not need this step. If there are other versions of figure files still present in your submission file inventory at resubmission, please replace them with the PACE-processed versions.

Reproducibility:

To enhance the reproducibility of your results, we recommend that authors of applicable studies deposit laboratory protocols in protocols.io, where a protocol can be assigned its own identifier (DOI) such that it can be cited independently in the future. Additionally, PLOS ONE offers an option to publish peer-reviewed clinical study protocols. Read more information on sharing protocols at https://plos.org/protocols?utm_medium=editorial-email&utm_source=authorletters&utm_campaign=protocols
